# Codon Usage and Phenotypic Divergences of SARS-CoV-2 Genes

**DOI:** 10.3390/v12050498

**Published:** 2020-04-30

**Authors:** Maddalena Dilucca, Sergio Forcelloni, Alexandros G. Georgakilas, Andrea Giansanti, Athanasia Pavlopoulou

**Affiliations:** 1 Physics Department, Sapienza University of Rome, 00185 Rome, Italy; sergio.forcelloni@gmail.com (S.F.); andrea.giansanti@oma1.infn.it (A.G.); 2 Liceo Scientifico Statale Augusto Righi, 00187 Rome, Italy; 3 DNA Damage Laboratory, Physics Department, School of Applied Mathematical and Physical Sciences, National Technical University of Athens (NTUA), Zografou Campous, 15780 Athens, Greece; alexg@mail.ntua.gr; 4 INFN Roma1 Unit, 00185 Rome, Italy; 5 Izmir Biomedicine and Genome Center (IBG), 35340 Balcova, Izmir, Turkey; athanasia.pavlopoulou@ibg.edu.tr; 6 Izmir International Biomedicine and Genome Institute, Dokuz Eylül University, 35340 Balcova, Izmir, Turkey

**Keywords:** coronaviruses, SARS-CoV-2, codon usage bias, mutational bias, natural selection, host adaptation

## Abstract

Severe acute respiratory syndrome coronavirus 2 (SARS-CoV-2), which first occurred in Wuhan (China) in December of 2019, causes a severe acute respiratory illness with a high mortality rate, and has spread around the world. To gain an understanding of the evolution of the newly emerging SARS-CoV-2, we herein analyzed the codon usage pattern of SARS-CoV-2. For this purpose, we compared the codon usage of SARS-CoV-2 with that of other viruses belonging to the subfamily of *Orthocoronavirinae*. We found that SARS-CoV-2 has a high AU content that strongly influences its codon usage, which appears to be better adapted to the human host. We also studied the evolutionary pressures that influence the codon usage of five conserved coronavirus genes encoding the viral replicase, spike, envelope, membrane and nucleocapsid proteins. We found different patterns of both mutational bias and natural selection that affect the codon usage of these genes. Moreover, we show here that the two integral membrane proteins (matrix and envelope) tend to evolve slowly by accumulating nucleotide mutations on their corresponding genes. Conversely, genes encoding nucleocapsid (N), viral replicase and spike proteins (S), although they are regarded as are important targets for the development of vaccines and antiviral drugs, tend to evolve faster in comparison to the two genes mentioned above. Overall, our results suggest that the higher divergence observed for the latter three genes could represent a significant barrier in the development of antiviral therapeutics against SARS-CoV-2.

## 1. Introduction

The name “coronavirus” is derived from the Greek κoρωνα, due to the viruses’ typical shapes being crown-like. The first complete genome of a coronavirus (mouse hepatitis virus—MHV), a positive sense, single-stranded RNA virus, was first reported in 1990 [1]. It belongs to the family *Coronaviridae* and ranges from 26.4 (ThCoV HKU12) to 31.7 (SW1) kb in genome length [2], having the largest genome among all known RNA viruses, with G + C contents varying from 32% to 43% [3]. The *Orthocoronavirinae* sub-family consists of four genera based on their genetic properties: *Alphacoronavirus*, *Betacoronavirus* (subdivided in subgroups A, B, C and D), *Gammacoronavirus* and *Deltacoronavirus*. Coronaviruses can infect humans and diverse animal species, including swine, cattle, horses, camels, cats, dogs, rodents, birds, bats, rabbits, ferrets, minks, snakes and other wildlife animals.

In this study, we have focused on 30 coronavirus (CoV) genomes: 28 viruses from Woo et al. (2010) [4]; the Middle East respiratory syndrome coronavirus (MERS-CoV), which appeared for the first time in 2012; and the severe acute respiratory syndrome coronavirus 2 (SARS-CoV-2), which just broke out in Wuhan (China) in December of 2019. Only seven CoVs have been identified that infect humans. Two coronaviruses that cause relatively mild respiratory symptoms have been known of since the 1960s that is, human CoV-229E (HCoV-229E) and human CoV-OC43 (HCoV-OC43). Human severe acute respiratory syndrome coronavirus (SARSr-CoV) was identified in 2003, and it causes a more severe respiratory syndrome [5]. The human coronavirus NL63 (HCoV-NL63) was first identified in 2004 and it causes respiratory symptoms in humans [6]; the fifth member, human CoV-HKU1 (HCoV-HKU1) was described in 2005 [7]. More recently, the pathogenic Middle East respiratory syndrome (MERS-CoV) coronavirus was identified as the sixth human coronavirus [8]. Finally, the present outbreak of a coronavirus-associated acute respiratory disease called coronavirus disease 19 (COVID-19) is caused by human SARS-CoV-2 infections [9,10].

The newly sequenced SARS-CoV-2 genome encodes two open reading frames (ORFs), ORF1a and ORF1ab. The latter encodes replicase polyproteins, and four structural proteins [11,12]; namely, the spike-surface glycoprotein (protein S), the small envelop protein (protein E), the matrix protein (M) and the nucleocapsid protein (N).

The phenomenon of codon usage bias (CUB) exists in many genomes, including RNA genomes, and it is actually determined by mutation and selection [13,14,15]. The non-random selection of synonymous codons is known to vary among species that are potential hosts for viruses [16]. It is therefore important to study patterns of common codon usage in coronaviruses because CUB can be related to the driving forces that shape the evolutions of small RNA viruses. Mutational bias has been considered as the major determinant of codon usage variation among RNA viruses [17]. Indeed, RNA viruses show an effective number of codons (ENC) that is quite high (ENC > 45), pointing to quite random codon usage, whereas the adaptive index CAI indicates that the viral CUB is consistent with that of the host, as observed in the Equine infectious anemia virus (EIAV) or Zaire ebolavirus (ZEBOV) [18].

The aims of this study were to perform a comprehensive analysis of the nucleotide composition, codon usage and rate of protein divergence of SARS-CoV-2, and to thereby draw inferences regarding its leading evolutionary determinants.

## 2. Materials and Methods

### 2.1. Sequence Data Acquisition

The complete coding genomic sequences of 306 isolates of SARS-CoV-2 reported across the world to date, were obtained from GISAID (available at https://www.gisaid.org/epiflu-applications/next-hcov-19-app/) and NCBI viral databases, accessed as of 17 March 2020. Then the sequences were selected according to their geographical distributions, isolation dates and host species.

In this study, we explored 30 CoV genomes: 28 viruses from Woo et al. (2010) [4]; the Middle East respiratory syndrome coronavirus (MERS-CoV); and the severe acute respiratory syndrome-related coronavirus 2 (SARS-CoV-2). We downloaded the coding sequences of these coronaviruses from the National Center for Biotechnological Information (NCBI) (available at https://www.ncbi.nlm.nih.gov/). For each virus, we investigated the following genes (shown in alphabetical order): *E, M, N, RdRP* and *S*.

### 2.2. Nucleotide Composition Analysis

The diverse nucleotide compositional properties were calculated for the coding sequences of the 30 CoV genomes. These compositional properties comprise the frequencies of occurrence of each nucleotide (A, U, G and C); AU and GC contents; and nucleotides G + C at the first (GC1), second (GC2) and third codon positions (GC3). To calculate these values, we used an in-house Python script. We calculated, also, the mean frequencies of nucleotides G + C at first and second positions (GC12).

### 2.3. RSCU

RSCU vectors for all the genomes were computed by using an in-house Python script, following the formula:

(1) $$RSCU_{i}=\frac{X_{i}}{\frac{1}{N_{i}}\sum_{j=1}^{n_{i}}X_{j}}$$

In the $RSCU_{i}$, $X_{i}$ is the number of occurrences in a given genome of codon i, and the sum in the denominator runs over its $n_{i}$ synonymous codons. If the RSCU value for a codon *i* is equal to 1, this codon has been chosen equally and randomly. Codons with RSCU values greater than 1 have positive codon usage bias, while those with a value less than 1 have relatively negative codon usage bias [19]. RSCU heat maps were drawn with the CIMminer software [20], which uses Euclidean distances and the average linkage algorithm.

### 2.4. Effective Number of Codons Analysis

ENC is an estimate of the frequency of different codons used in a coding sequence. In general, ENC ranges from 20 (when each amino acid is coded by the same codon) to 61 (when all synonymous codons are used on an equal footing). Given a sequence of interest, the computation of ENC starts from $F_{\alpha }$, a quantity defined for each family α of synonymous codons (one for each amino acid):

(2) $$F_{\alpha }={\left(\frac{n_{k\alpha }}{n_{\alpha }}\right)}^{2}$$

where $m_{\alpha }$ is the number of different codons in α (each one appearing $n_{1_{\alpha }},n_{2_{\alpha }},\ldots ,n_{m_{\alpha }}$ times in the sequence) and $n_{\alpha }=\sum_{k=1}^{m_{\alpha }}n_{k_{\alpha }}$.

ENC then weights these quantities on a sequence:

(3) $$ENC=N_{s}+\frac{K_{2}\sum_{\alpha =1}^{K_{2}}n_{\alpha }}{\sum_{\alpha =1}^{K_{2}}\left(n_{\alpha }F_{\alpha }\right)}+\frac{K_{3}\sum_{\alpha =1}^{K_{3}}n_{\alpha }}{\sum_{\alpha =1}^{K_{3}}\left(n_{\alpha }F_{\alpha }\right)}+\frac{K_{4}\sum_{\alpha =1}^{K_{4}}n_{\alpha }}{\sum_{\alpha =1}^{K_{4}}\left(n_{\alpha }F_{\alpha }\right)}$$

where $N_{S}$ is the number of families with one codon only and $K_{m}$ is the number of families with degeneracy *m* (the set of 6 synonymous codons for Leu can be split into one family with degeneracy 2, similar to that of Phe, and one family with degeneracy 4, similar to that, e.g., of Pro). ENC was evaluated by using the implementation in DAMBE 5.0 [21].

### 2.5. Codon Adaptation Index

The codon adaptation index CAI [22] was used to quantify the codon usage similarities between the virus and host coding sequences. The principle behind CAI is that codon usage in highly expressed genes can reveal the optimal (i.e., most efficient for translation) codons for each amino acid. Hence, CAI is calculated based on a reference set of highly expressed genes to assess, for each codon *i*, the relative synonymous codon usages ($RSCU_{i}$) and the relative codon adaptiveness ($w_{i}$):

(4) $$RSCU_{i}=\frac{X_{i}}{\frac{1}{n_{i}}\overset{n_{i}}{\sum_{j=1}}X_{j}};\;\;w_{i}=\frac{RSCU_{i}}{\max_{j=1,\ldots ,n_{i}}\;\left\{RSCU_{j}\right\}};$$

In the $RSCU_{i}$, $X_{i}$ is the number of occurrences of codon *i* in the genome, and the sum in the denominator runs over the $n_{i}$ synonyms of *i*; RSCUs thus measures codon usage bias within a family of synonymous codons. Then $w_{i}$ is then defined as the usage frequency of codon *i* compared to that of the optimal codon for the same amino acid encoded by *i*—(i.e., the one which is mostly used in a reference set of highly expressed genes). The CAI for a given gene *g* is calculated as the geometric mean of the usage frequencies of codons in that gene, normalized to the maximum CAI value possible for a gene with the same amino acid composition:

(5) $$CAI_{g}={\left(\overset{l_{g}}{\prod_{i=1}}w_{i}\right)}^{1/l_{g}},$$

where the product runs over the $l_{g}$ codons belonging to that gene (except the stop codon).

This index values range from 0 to 1, where the score 1 represents the tendency of a gene to use the most frequently used synonymous codons in the host. The CAI analysis of these coding sequences is performed using DAMBE 5.0 [21]. The synonymous codon usage data of different hosts (human and other species) were retrieved from the codon usage database (http://www.kazusa.or.jp/codon/).

To study the patterns of codon biases in the coronaviruses, we used Z-score values:

(6) $$Z_{v}\left[\left(ENC\right)\right]=\frac{{\langle ENC\rangle }_{CoV}-{\langle ENC\rangle }_{v}}{\sigma_{v}/\sqrt{N_{v}}},$$

where ${\langle ENC\rangle }_{CoV}$ is the average of the ratio within a codon bias index in a coronavirus *v*, ${\langle ENC\rangle }_{v}$, and $\sigma_{v}$ is the average value of ENC and its standard deviation over the whole virus *v*; and $N_{v}$ is the number of viruses (we use the standard deviation of the mean when comparing average values).

The same Z-score was evaluated for codon bias index CAI.

### 2.6. The Similarity Index

The similarity index (SiD) provides a measure of similarity in codon usage between the virus (in our case, SARS-CoV-2) and the host under study. Formally, it is defined as follows:

(7) $$R\left(a,b\right)=\frac{\sum_{k=1}^{59}a_{i}\cdot b_{i}}{\sqrt{\sum_{k=1}^{59}a_{i}^{2}\cdot \sum_{k=1}^{59}b_{i}^{2}}}$$

(8) $$SiD=\frac{1-R(a,b)}{2}$$

where $a_{i}$ is the RSCU value of 59 synonymous codons of the SARS-CoV-2 coding sequences; $b_{i}$ is the RSCU value of the identical codons of the potential host. *R*(*a*,*b*) is defined as the cosine value of the angle included between A and B spatial vectors, and therefore, quantifies the degree of similarity between the virus and the host in terms of their codon usage patterns. In our analysis, we considered the hosts species shown in Table 1 by Woo et al. [4]. We also considered snakes and pangolins, because they were previously identified as possible candidates for the novel coronavirus spillover into humans [9]. SiD values range from 0 to 1. Specifically, the higher the value of SiD, the more adapted the codon usage of SARS-CoV-2 to the host [23].

### 2.7. ENC Plot

ENC-plot analysis was performed to estimate the relative contributions of mutational bias and natural selection in shaping CUB of genes encoding proteins that are crucial for SARS-CoV-2: RdRP, the spike-surface glycoprotein (protein S), the small envelop protein (protein E), the matrix protein (M) and the nucleocapsid protein (N). The ENC-plot is a plot in which ENC is the ordinate and the GC-content in the third codon position (GC3) is the abscissa. Depending on the action of mutational bias and natural selection, different cases are discernable. If a gene is not subject to selection, a clear relationship is expected between ENC and GC3 [24]:

(9) $$ENC=2+s+\frac{29}{s^{2}+{\left(1-s\right)}^{2}}$$

where *s* represents the value of GC3 [24]. For those genes, codon preference, determined only by mutational bias, is expected to lie on or just below Wright’s theoretical curve. Alternatively, if a particular gene is subject to selection, then it falls below Wright’s theoretical curve. In this case, the vertical distance between the point and the theoretical curve provides an estimation of the relative extent to which natural selection and mutational bias affect CUB.

To evaluate the dots scattering from Wright’s theoretical curve, we calculated the module of distance, and the box plots were drawn with an in-house Python script.

### 2.8. Neutrality Plot

We performed neutrality plot analysis [25] to estimate the relative contributions of natural selection and mutational bias in shaping the CUBs of five crucial coronavirus genes in the research field aiming to develop a vaccine against SARS-CoV-2: *M, N, S, RdRP* and *E*. In this analysis, the GC1 or GC2 values (ordinate) were plotted against the GC3 values (abscissa), and each gene was represented as a single point on this plane. In this case, the three stop codons (UAA, UAG and UGA) and the three codons for isoleucine (AUU, AUC and AUA) were excluded from the calculation of GC3, and two single codons for methionine (AUG) and tryptophan (UGG) were excluded in all three (GC1, GC2 and GC3) [25].

For each gene, we separately performed a Spearman correlation analysis between GC1 and GC2 with the GC3. If the correlation between GC12 and GC3 is statistically significant, the slope of the regression line provides a measure of the relative extent to which natural selection and mutational bias affect the CUBs of these genes (Sueoka 1999). In particular, if the mutational bias is the driving force that shapes the CUB, then the corresponding data points should be distributed along the bisector (slope of unity). On the other hand, if natural selection also affects the codon choice of a family of genes, then the corresponding regression line should diverge from the bisector. Thus, the divergence between the regression line and bisector quantifies the extent of codon usage preference due to the natural selection.

### 2.9. Forsdyke Plot

To study the mutational rates of genes *M, N, S, RdRP* and *E*, we performed an analysis by using our previously defined Forsdyke plot [26]. Each gene in SARS-CoV-2 (used as a reference) was compared to its orthologous gene in the 30 coronaviruses considered in this analysis. Each pair of orthologous genes is represented by a point in the Forsdyke plot, where protein divergence is correlated with DNA divergence (see Methods in [26] for details). The protein sequences were aligned using Biopython. The DNA sequences were then aligned using the protein alignments as templates.

Then, both DNA and protein divergences were assessed as explained in Methods in [26] by counting the number of mismatches in each pair of aligned sequences. Thus, each point in the Forsdyke plot measures the divergence between pairs of orthologous genes in the two species, as projected along with the phenotypic (protein) and nucleotidic (DNA) axis. The first step in each comparison is to compute the regression line between protein vs. DNA sequence divergence in the Forsdyke plot getting values of intercept and slope for each variant of genes (i.e., *M, N, S, RdRP* and *E*). To test whether the regression parameters associated with each variant are different or not, we followed a protocol founded by Dilucca et al., considering a *p*-value ≤ 0.05.

### 2.10. Phylogenetic Analysis

To explore the evolutionary relationships among the four genera of coronaviruses, phylogenetic analysis of the full-length genomic sequences of the 30 CoVs listed in Table 1 was performed. The sequences were aligned with the usage of ClustalO [27,28]. The resulting multiple sequence alignment was used to build a phylogenetic tree by employing a maximum likelihood (ML) method implemented in the software package MEGA version 10. 1 [29]. ModelTest-NG [30] was used to select the best-fit evolutionary model of nucleotide substitution; that is, GTR + G + I. Bootstrap analysis (100 pseudo-replicates) was conducted in order to evaluate the statistical significance of the inferred trees.

## 3. Results

### 3.1. Nucleotide Composition

We calculated the nucleotide compositions of the coronavirus genomes under study (see Table 1). Previous results showed that the gene N, which follows the trend A > U > G > C [12] and the coronavirus RNA genomes are biased towards high AU content and low GC content [31]. In line with that, our results show that the nucleotide A is the most frequent base and the nucleotide composition follows the trend A > U > G > C (see Table 2). Interestingly, SARS-CoV-2 has a nucleotide composition that is similar to the other CoVs but with a different trend U > A > G > C. The GC content in SARS-CoV-2 is 0.37 ± 0.05.

### 3.2. All the Sequenced SARS-CoV-2 Genomes Share a Common Codon Usage

**Figure A1:**
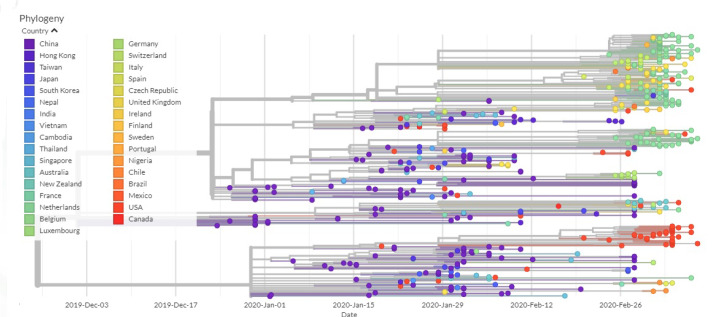
Phylogenetic tree from GISAID.

**Figure A2:**
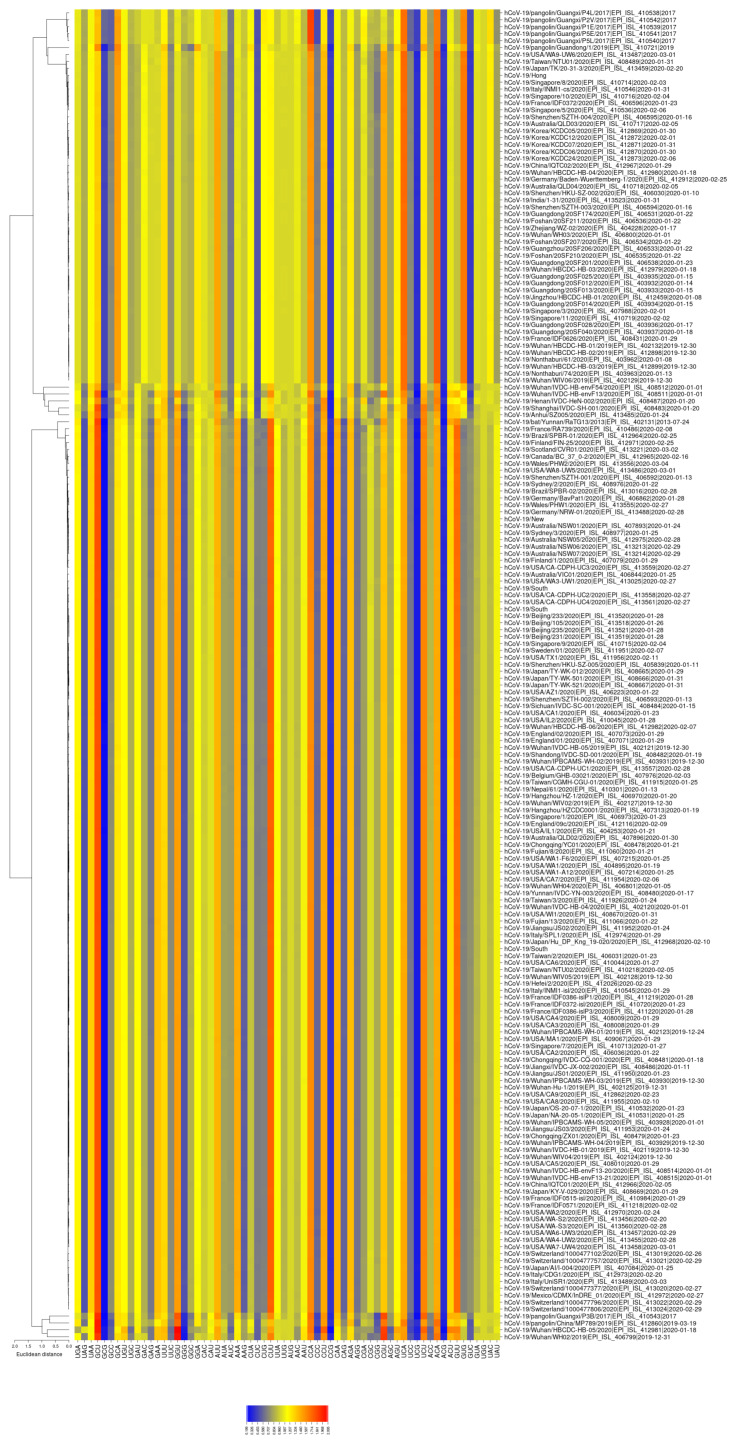
**RSCU vectors of coronavirus.** Patterns of RSCU vectors for 306 patients with SARS-CoV-2 from different countries (data downloaded from GISAID).

We downloaded the protein-coding sequences of SARS-CoV-2 from GISAID database, and classified each SARS-CoV-2 based on the geographic location in which it was sequenced (see tree in Figure A1). For each SARS-CoV-2 genome, we calculated the relative synonymous codon usage (RSCU), in the form of a 61-component vector. The heatmap and the associated clustering of these vectors are shown in Figure A2. We noted that the overall codon usage bias among SARS-CoV-2 strains appears to be similar. Moreover, their associated RSCU vectors did not cluster according to geographic location, thereby confirming the common origin of these genomes. Motivated by these observations, we considered a unique vector to represent the codon usage of SARS-CoV-2 in the following analyses.

### 3.3. Codon Usage of SARS-CoV-2

**Figure 1:**
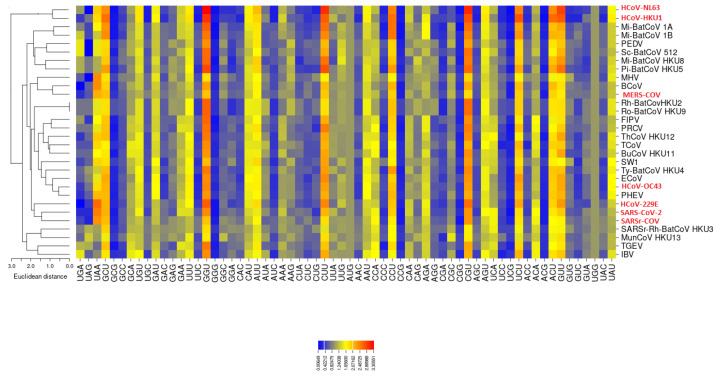
**Clustering of the relative synonymous codon usage (RSCU) vectors associated with the 30 coronaviruses.** Human coronaviruses are shown in red. The newly identified SARS-CoV-2 coronavirus is closer to HCoV-229E and SARSr-CoV in terms of codon usage, as measured by their RSCU vectors. Heatmap was drawn with the CIMminer software [20], which uses Euclidean distances and the average linkage algorithm.

We compared the codon usage of SARS-CoV-2 with that of the other coronavirus genomes. For this purpose, we used the RSCU, which is a biologically relevant metric of the distance between the codon usage in the protein-coding sequences of these genomes. The heatmap of the RSCU values associated with the coronaviruses is shown in Figure 1. The RSCU values of the majority of the codons scored between 0 and 3.1 (see legend in Figure 1). Interestingly, the newly identified SARS-CoV-2 Wuhan-Hu-1 coronavirus clusters with the other two human coronaviruses SARSr-CoV and HCoV-229E. Moreover, in this heatmap, HCoV-HKU1 and HCoV-NL63 cluster together, consistent with viral adaptation to their host.

In line with previous observations, we show that the mean CpG relative abundance in the coronavirus genomes is markedly suppressed [32]. Specifically, GGG, GGC, CCG (pyrimidine-CpG) and ACG (purine-CpG) present low frequencies of occurrence, probably due to the relative tRNA abundance of the host. In SARS-CoV-2, the most frequently used codons are CGU (arginine, 2.34 times) and GGU (glycine, 2.42), whereas the least used codons are GGG (glycine) and UCG (serine). Of note, the most frequently used codons for each amino acid end with either U or A [18].

### 3.4. The Codon Usage of SARS-CoV-2 in Relation to the Human Host

To measure the codon usage bias in the coronavirus genomes, we used the effective number of codons (ENC) and the competition adaptation index (CAI). For each coronavirus, we calculated the average values of CAI and ENC associated with its genes. In Table 3 the ENC and CAI values for all the coronaviruses considered in this work are reported. To visually enhance the differences among the codon usage of these coronaviruses, we calculated the Z-score value of each virus with respect to the average values of ENC and CAI calculated for all 30 coronaviruses.

**Figure 2:**
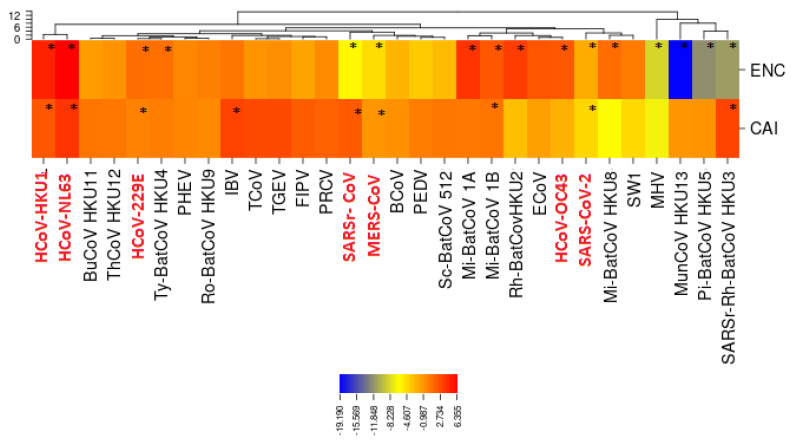
**Z-score values.** Z-score is calculated for two codon bias indeces: effective number of codons (ENC) and the competition adaptation index (CAI). CAI values are calculated by considering the hosts specified in Table 3 by Woo et al. [4]. Regarding SARS-CoV-2, we considered a human host. In red, we show the human coronaviruses. Several coronaviruses have a codon usage preference values higher than the average value of the family (|Z-score| > 3). The statistically significant differences are marked with asterisks. In particular, SARS-CoV-2 genes have average values of CAI and ENC that are higher than the average of all coronaviruses. (*): |Z-score| > 3.

The human coronaviruses show different patterns of codon usage (Figure 2). With the exception of HCoV-OC43, all the human coronaviruses have ENC and CAI values that are significantly different from the average values of ENC and CAI calculated for all 30 coronaviruses (|Z-score| > 3). Specifically, the ENC value associated with SARS-CoV-2 (51.9 ± 2.59) is significantly higher than the average of all coronaviruses (50.09 ± 1.32), indicating that SARS-CoV-2 uses a broader set of synonymous codons in its coding sequences. Moreover, the CAI of SARS-CoV-2 (0.727 ± 0.054) is markedly higher than the average one (0.69 ± 0.024), underscoring that SARS-CoV-2 uses codons that are better adapted to its host. Moreover, the CAI of SARS-CoV-2 is significantly higher than the CAI of the other human CoVs in the subfamily, thereby suggesting a greater adaptation to the human host for SARS-CoV-2 compared to the other coronaviruses. Finally, the ENC values of the three most pathogenic HCoVs having Z-scores > 3 (SARS-CoV, SARS-CoV-2 and MERS) are on average, higher than the ENCs of the other four HCoVs, which have instead Z-scores <−3. This higher CUB in terms of ENCs of the four HCoVs reinforces their strong adaptiveness to humans, as they have been circulating in the population for a long time and are now less pathogenic.

**Figure 3:**
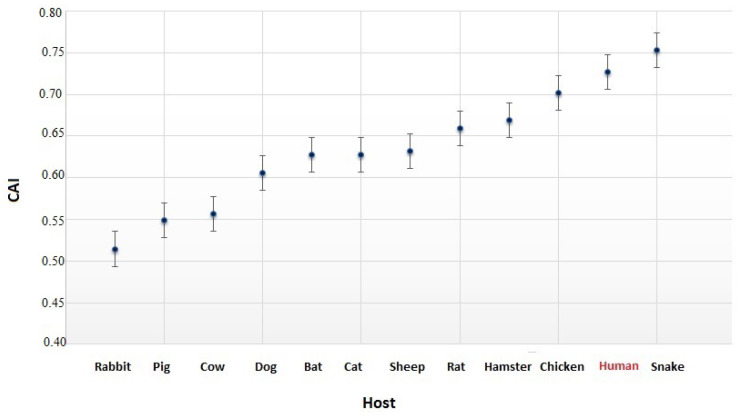
**CAI values of SARS-CoV-2 for different hosts.** On the horizontal axis, the 12 eukaryotic species are shown that were considered in the comparisons. The host species are ranked in ascending order. CAI values for snake and human hosts are higher than those for other hosts.

To better clarify the origin of SARS-CoV-2 and its optimization to the human host, we then calculated the average CAI for the SARS-CoV-2 genes by using different reference hosts (Figure 3).

Interestingly, snake and human hosts correspond to the highest values of CAI, indicating that SARS-CoV-2 uses codons that are better optimized to these two organisms. Although our results suggest a possible origin of SARS-CoV-2 from snakes and its spillover into humans [33], previous studies do not support this hypothesis [34,35].

Similarly, to corroborate this observation, we also calculated the similarity index (SiD) of SARS-CoV-2 for the hosts reported in Figure 3 (see Figure A4). SiD values range from 0 to 1; the higher the value of SiD, the more adapted the codon usage of SARS-CoV-2 to the host [23]. Since recent studies have revealed multiple lineages of Malayan pangolin (*Manis javanica*) coronavirus that are similar to SARS-CoV-2 [36], we also added this organism in the present analysis. CAI was not calculated for pangolin because its genome is not well-annotated, and the five genes under investigation (M, N, S, E and RdRp) are not available. SiD values range from 0.23 (in rabbit) to 0.78 (in human). Notably, this analysis not only confirms our previous observation (see Figure 3) that SARS-CoV-2 uses codons that are better optimized to snakes (SiD = 0.75) and humans (SiD = 0.78), but reveals the same for pangolins (SiD = 0.76), bats (SiD = 0.70 ), and rats (SiD = 0.71), which are also possible hosts for SARS-CoV-2 [9].

### 3.5. Selective Pressures and Mutational Rates Characterizing Five Conserved Coronavirus Genes

The genome of the newly emerging SARS-CoV-2 consists of a single, positive-stranded RNA, which is approximately 30,000 nucleotides long. The newly sequenced SARS-CoV-2 genome is organized similarly to the other coronavirus genomes. Ceraolo et al. performed a cross-species analysis for all proteins encoded by SARS-CoV-2 (see Figure 3 and Figure 4 in [37]). It encodes polyproteins common to all betacoronaviruses which are further cleaved into the individual structural proteins E, M, N and S, and the non-structural RdRP [38]. Thus, only five viral genes, classified according to their viral locations, were studied for each virus, because the short length and insufficient codon usage diversity of the other genes might have biased our results.

**Figure 4:**
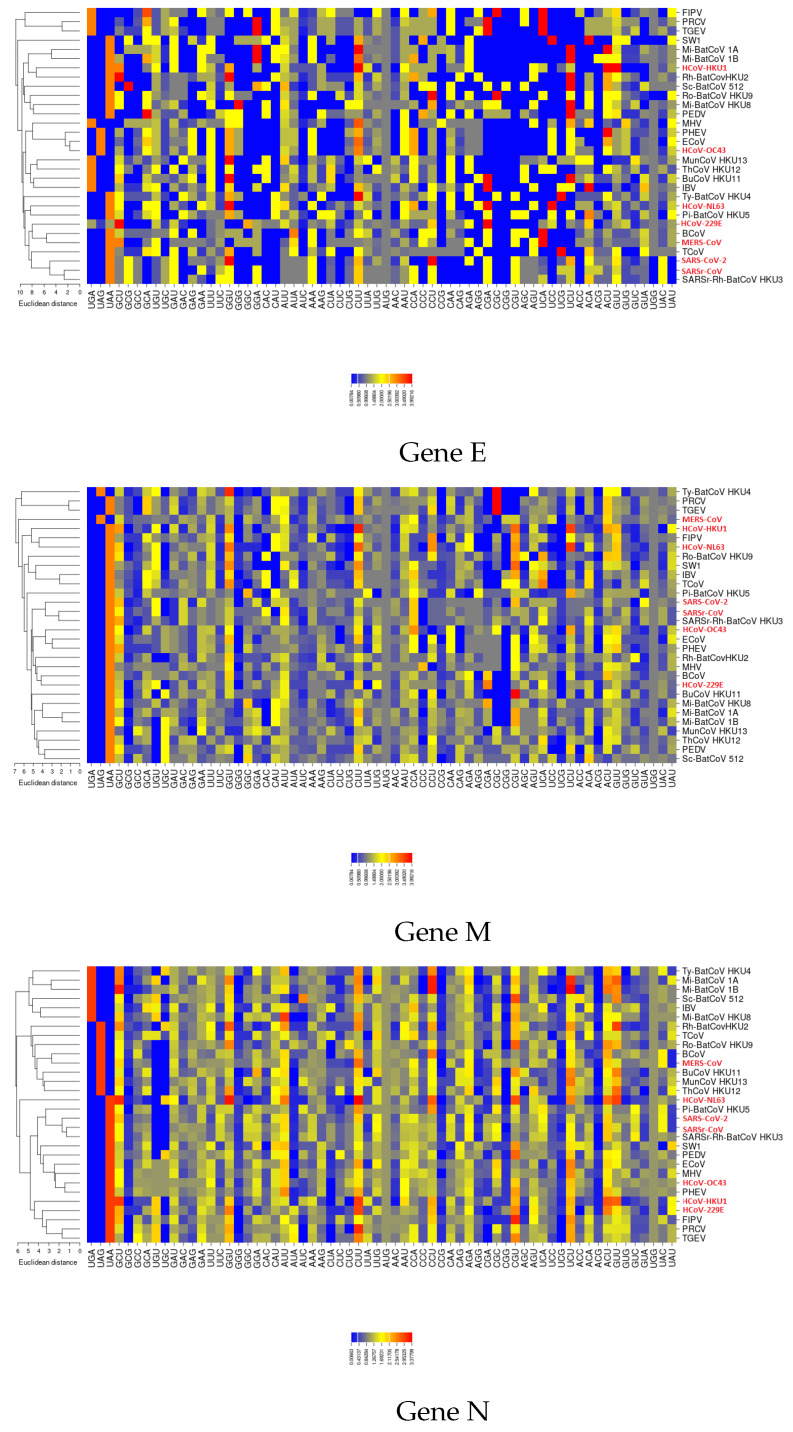
**RSCU vectors of three different coronavirus genes.** Heatmaps confirm that the RSCU patterns of the newly identified coronavirus SARS-CoV-2 sequence are more related to those of SARSr-CoV and SARSr-Rh-BatCoV HKU3 for genes *E, M* and *N*.

**Figure A3:**
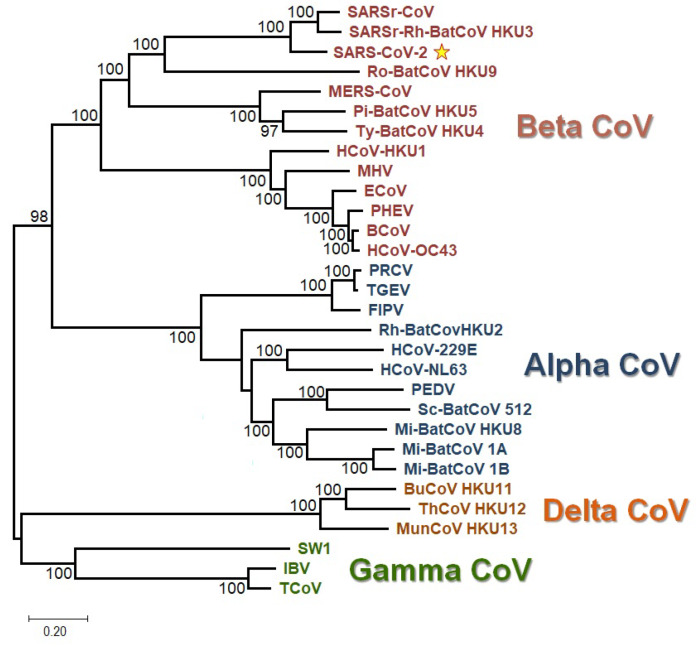
**Unrooted ML-based tree of the 30 CoV genomic sequences.** The four distinct color-coded clades correspond to the respective genera of CoVs. The SARS-CoV-2 sequence is indicated by a star. The branch lengths depict evolutionary distance. Bootstrap values higher than 50 are shown at the nodes. The scale bar at the lower left denotes the length of nucleotide substitutions per position.

**Figure A4:**
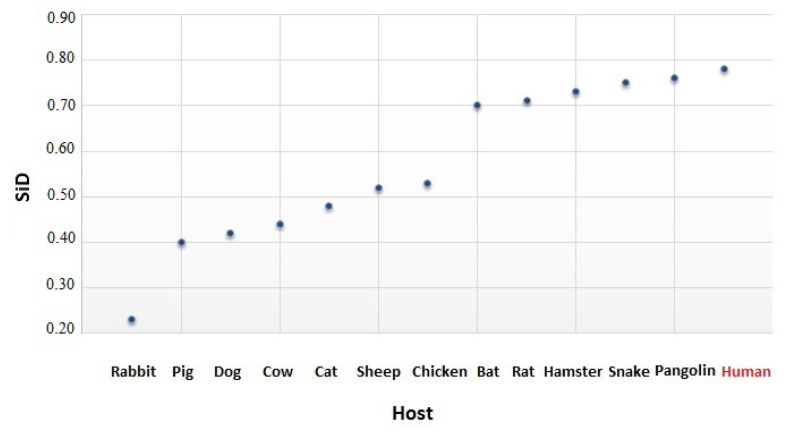
**Similarity index (SiD) of SARS-CoV-2, using different host organisms as references.** On the horizontal axis, the 13 eukaryotic species that were considered in the comparisons are shown. The host species are ranked in ascending order. CAI values for the bat, rat, hamster, snake, pangolin, and human are higher compared to the other species.

**Figure A5:**
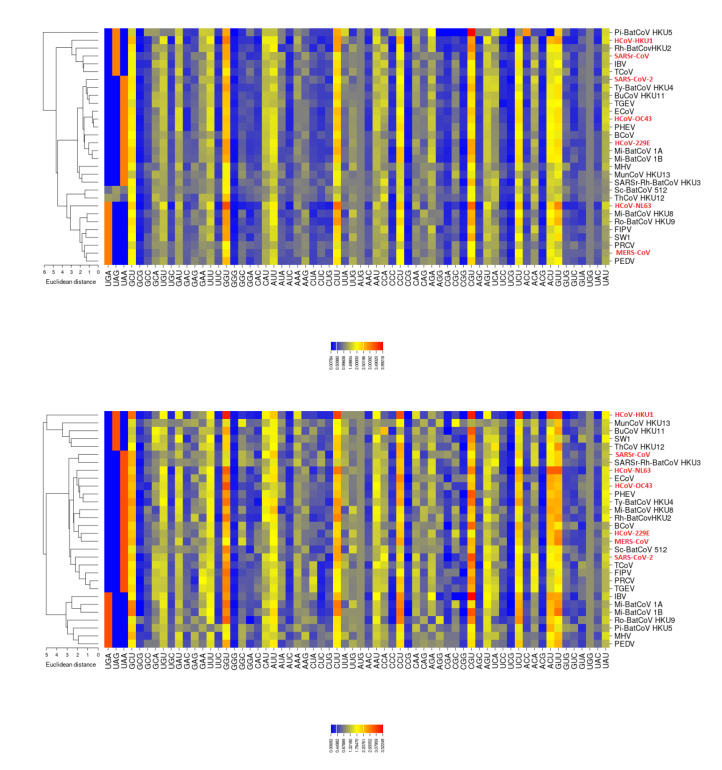
Heatmaps of RSCU vectors for genes *RdRP* (**upper panel**) and *S* (**bottom panel**).

The corresponding gene products are involved in essential viral functions. Briefly, S protein regulates viral attachment to the receptor of the target host cell [39]; E protein functions to assemble the virions and acts as an ion channel [40] M protein plays a role in viral assembly and is involved in the biosynthesis of new virus particles [41]; N protein forms the ribonucleoprotein complex with the viral RNA [12]; RdRP catalyzes viral RNA synthesis. For these five proteins the RSCU vectors in each virus of the dataset are shown in Figure 4 and Figure A5. We showed that SARS-CoV-2 clusters with SARSr-CoV and SARSr-Rh-BatCoV HKU3, only for genes *E, M* and *N*, consistent with the inferred phylogeny shown in Figure A3.

### 3.6. The ENC Plot Analysis of Individual Genes of SARS-CoV-2

**Figure 5:**
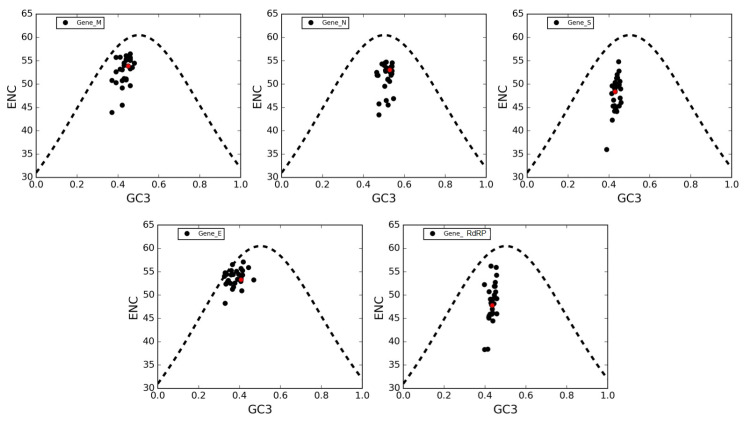
**ENC-plots of genes *M, N, S, E* and *RdRP*.** In these plots, each point corresponds to a single gene. The black-dotted lines in all panels are plots of Wright’s theoretical curve corresponding to codon usage biases (CUBs) that occur merely due to mutational bias (no selective pressure). Red dots represent SARS-CoV-2 genes.

**Figure 6:**
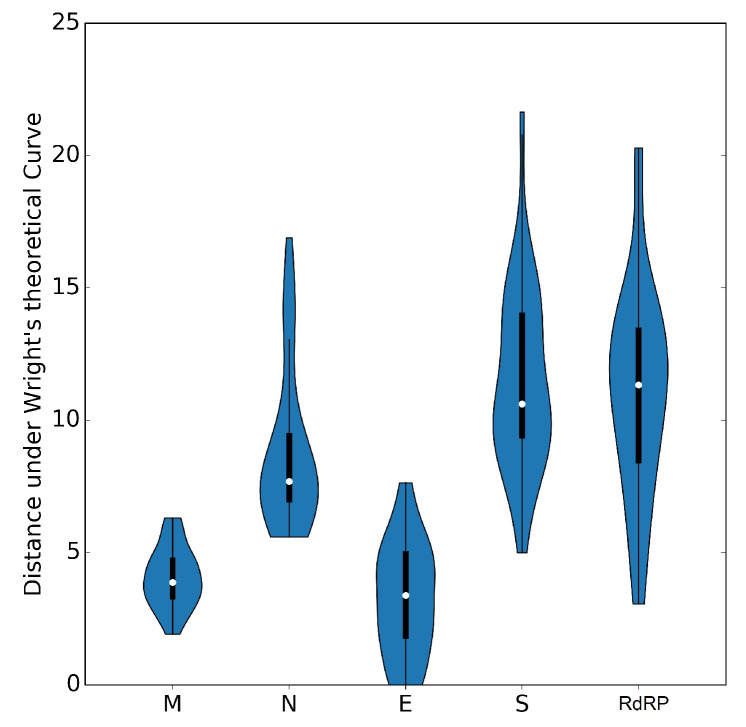
Violin plots of the distances of genes *M, N, S, E* and *RdRP* from Wright’s theoretical curve.

To further investigate which factors account for the low codon usage bias of the coronavirus genes, we analyzed the relationship between the ENC value and the percentage of G or C in the third codon position (GC3s). The ENC-plots obtained for the five genes (*M, N, S, E* and *RdRP*) are shown separately together with Wright’s theoretical curve (Figure 5), denoting that GC3s is only determined exclusively by codon usage [24]. Thus, if mutational bias, as quantified by GC-content in the generally neutral third codon position, is the main factor in determining the codon usage among these genes, the corresponding point in the ENC-plot should lie on or just below Wright’s curve. In Figure 5, all distributions lie below the theoretical curve, an indication that not only mutational bias but also natural selection play non-negligible roles in the codon choices in all genes. This is also exemplified by the violin plots in Figure 6 showing the distances between the genes and Wright’s theoretical curve in the ENC-plot.

Genes *N, S* and *RdRP* are more scattered below the theoretical curve than genes *M* and *E*, implying that in the latter the codon usage patterns are pretty consistent with the effects of mutational bias. Interestingly, data points corresponding to the gene *N*, which is the major viral structural component needed to protect and encapsidate the viral RNA, are clustered more closely around GC3 = 0.5 (see Figure 5). This means that the displacement under Wright’s theoretical curve most likely reflects the selective pressure exerted on this gene. Conversely, all other genes show a displacement towards lower values of GC3-content, thereby corroborating our previously mentioned observation that coronaviruses tend to use codons that end with A and U (see Section 3.3).

### 3.7. Neutrality Plot of Individual Genes of SARS-CoV-2

**Figure 7:**
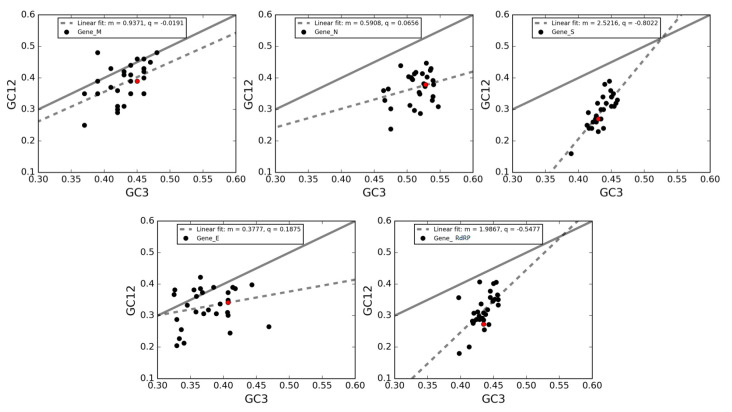
**Neutrality plot of genes *M, N, S, E* and *RdRP*.** In these plots, each point corresponds to a single gene in a virus. The solid black lines in all panels are the bisectors corresponding to those CUBs occurring merely due to mutational bias (no selective pressure). The black-dotted lines are the linear regressions. Red dots represent SARS-CoV-2 genes.

A neutrality plot analysis was performed to estimate the role of mutational bias and natural selection in shaping the codon usage patterns of the five genes under investigation. In this plot, the average GC-content in the first and second positions of codons (GC12) is plotted against GC3s, which is considered as a pure mutational parameter. In Figure 7, the neutrality plots obtained for genes *M, N, S, E* and *RdRP*, together with the best-fit lines and the slopes associated with them are shown.

To understand the rationale behind these results: the wider the deviation between the slope of the regression line and the bisector, the stronger the action of selective pressure. All correlations are highly significant (Spearman correlation—$R^{2}$ analysis, *p*-value < 0.0001). By comparing the divergences between the regression lines and the bisectors in each panel, we reveal that the five genes considered herein depend on a balance between natural selection and mutational bias.

Specifically, in line with the ENC-plot analyses, the genes *S* and *RdRP* present the largest deviations of their regression lines from the bisector lines, thereby indicating a stronger action of natural selection. Conversely, the regression line for the gene *M* is closer to the bisector than the other genes, meaning that this gene is the least one subject to the action of natural selection. Finally, the genes *E* and *N* are intermediate between the previous cases.

Notably, almost all data points are clustered below the bisector lines, implying a selective tendency for a higher AU content in the first two codon positions than in the third one. Additionally, both GC3 and GC12 are lower than 0.5, reflecting a general preference for A and U bases in all three codon positions. Interestingly, data points associated to gene *M* and *E* are closer to the bisector lines compared to genes *N, S,* and *RdRP*. Based on this observation, we could suggest that the GC content in the first two codon positions tends to be in proportion to GC3 in genes *M* and *E*, and this partially explains the closeness of these two genes to the Wright theoretical curve in Figure 5.

### 3.8. Forsdyke Plot of Individual Genes of SARS-CoV-2

**Figure 8:**
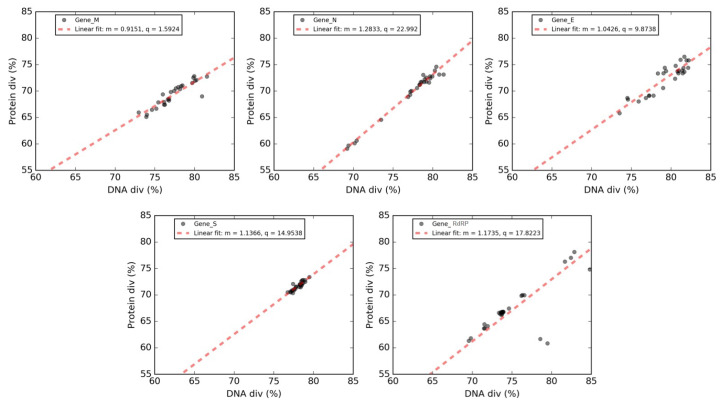
**Forsdyke plots of genes *M, N, S, E* and *RdRP*.** Phenotype (Protein div) vs. nucleotide (DNA div) sequence divergence between SARS-CoV-2 and orthologous genes in the other coronaviruses. Each point corresponds to an individual gene. In each panel, the best-fit line is shown in red, together with the associated values of the slope (m) and the intercept (q) in the legend.

We analyzed the DNA divergence and protein sequence divergence that characterize these five genes by comparing the nucleotide sequences of the newly emerging SARS-CoV-2 and their corresponding protein sequences with those of other coronaviruses under study. Each *SARS-CoV-2* gene was compared to its orthologous gene in the 30 coronaviruses to estimate evolutionary divergences. Each pair of orthologous genes is represented by a point in the Forsdyke plot [26], where protein divergences correlated with DNA divergence. Each point in the Forsdyke plots measures the divergence between pairs of orthologous genes in the two species, as projected along with the phenotypic (protein) and nucleotide (DNA) axis. Thus, the slope is an estimation of the fraction of DNA mutations that result in amino acid substitutions [26]. In Figure 8, a separate Forsdyke plot is shown for each gene.

Overall, protein and DNA sequence divergences are linearly correlated, and these correlations correspond to slopes and intercepts of the regression lines.

Genes *M* and *E* display quite low slopes, indicating that these proteins tend to evolve slowly by accumulating nucleotide mutations on their corresponding genes. Conversely, the steeper slopes for genes *N, RdRP* and *S* suggest that these genes tend to evolve faster compared to other ones. A plausible explanation for this observation is that protein N, due to its immunogenicity, has been frequently used to generate specific antibodies against various animal coronavirus, including SARS [42]. The viral replicase polyprotein is essential for the replication of viral RNA, and finally, gene S encodes the protein that is responsible for the “spikes” present on the surface of coronaviruses. Our results suggest that the higher divergence observed in these three proteins could represent a major obstacle to the development of an therapeutic treatment against SARS-CoV-2.

## 4. Discussion

To investigate the factors determining the codon usage patterns of SARS-CoV-2 and other coronaviruses, several analytical methods were used in our study. First, the RSCU value of the SARS-CoV-2 was calculated. Despite the relatively high mutation rate that characterizes SARS-CoV-2, as other RNA viruses, we could not find any significant differences in codon usage between its genome and the ones of the other CoVs. Moreover, their associated vectors did not cluster based on geographical position, further confirming the common origin of these genomes.

In line with the common nucleotide composition of other RNA viruses such as SARS, our results show that SARS-CoV-2 has a high AU content and a low GC content. The results also indicate that codon usage bias exists and that SARS-CoV-2 prefers U-ending codons. The codon usage bias was further confirmed by a mean ENC value of 51.9 (a value greater than 45 is considered a slight codon usage bias due to mutation pressure or nucleotide compositional constraints). These findings were also corroborated by the CAI analysis, which measures the deviation of a given protein coding gene sequence with respect to a reference set of the most highly expressed genes in the host. This suggests that those RNA viruses with high ENC values (and low CAI) adapt to the host with randomly chosen codons. Therefore, a slightly biased codon usage pattern might allow the virus to use several codons for a respective amino acid, and it might be beneficial for viral replication and translation in host cells.

We then analyzed in more detail the relationships between SARS-CoV-2 and various possible hosts other than humans. For this purpose, we calculated the average CAI and SiD values of individual SARS-CoV-2 genes against different candidate hosts. Although previous studies do not support transmission of SARS-CoV-2 from snakes to humans [34,35], we showed that SARS-CoV-2 has the highest CAI values by considering these two organisms as references, and therefore, it should use codons that are better optimized to snakes and humans. Moreover, we demonstrated that the adaptiveness of SARS-CoV-2’s codon usage, as measured by SiD, is also fairly high for pangolins, rats, and bats, thereby confirming previous hypotheses regarding the possible origin of SARS-CoV-2 from these species [9].

The ENC-plot analysis indicated that natural selection plays an important role in the codon choice of the five conserved viral genes under study; namely, *RdRP*, *S*, *E*, *M* and *N*. However, genes *N*,*S* and *RdRP* are more scattered below the theoretical curve compared to genes *M* and *E*, implying that in the latter the codon usage is more a sign of mutational bias than of natural selection. According to neutrality plot analysis, the genes *S* and *RdRP* are considered to be subject to more robust action of natural selection; gene *M* is the least subject to natural selection; and the genes *E* and *N* are in an intermediate situation. Conversely, the regression line for the gene *M* is closer to the bisector than the other genes, meaning that this gene is the least subject to the action of natural selection. Finally, the genes *E* and *N* are intermediately affected regarding the previous cases.

Forsdyke plots were employed to analyze the mutation statuses of these five genes. Proteins M and E were found to have gentler slopes, thereby reflecting a tendency to evolve slowly by accumulating nucleotide mutations on their respective genes. Conversely, the steeper slopes for the three genes *N, RdRP* and *S* (encoding a protein responsible for the “spikes” present on the surface of coronaviruses), indicate that these three genes, and therefore their corresponding protein products, evolve faster compared to the other two genes.

Interestingly, all x-intercepts (see Table 4) are negative and the degree of negativity correlates with the low slope values. Recalling that the x-axis (RNA change) can be viewed as a time axis, it appears that the RNA segments encoding *M* and *E* are as resistant to change during the early period of genome divergence (negative x values) as they are during the later period of divergence when phenotypic changes can be naturally selected (positive x values). M and E are less flexible at the protein level. On the other hand, the RNA segments encoding S, RdRP and N are flexible during the early genome divergence period (high negative x values). As a result, these segments would have been more able to contribute to the initial genotypic divergence that would have decreased recombination between two genomes diverging in a common cell, thereby facilitating speciation. Under the protection of this global “reproductive isolation”, the segments could then evolve during the period corresponding to positive x values. Without reproductive isolation, blending would have occurred and phenotypic divergence would be less possible.

In future studies, it would be interesting to explore why *M* and *E* are less flexible and *S, R* and *N* are more flexible towards preventing recombination. Viral RNA recombination requires recognition between two comparable RNA regions and then extensive base pairing, mediated by the kissing stem-loop interaction, to thoroughly examine sequence complementarity. Perhaps the *M* and *E* genes lack the ability to form stem-loops, but this inflexibility during phenotypic divergence is suggestive of high conservation.

The findings of the present study could be useful for developing diagnostic reagents and probes for detecting a wide range of viruses and isolates in one test and for vaccine development, utilizing the information about codon usage patterns in these genes.

In addition, an interesting potential idea for the treatment of pneumonia-related to SARS-CoV-2 and other similar viruses is a low dose of ionizing radiation (LDIR). SARS-COV-2 is an RNA virus with an expected mutation rate similar to other RNA viruses, as discussed above. This mutation rate is usually much higher than the corresponding one of any human host. Therefore, as discussed in a recent paper [43], any antiviral drug against SARS-CoV-2 would exert an intense selective pressure on the virus. This may result in highly adaptive and treatment-resistant virus types with enhanced pathogenicity. It should also be taken into consideration that the virus will create a systemic inflammatory response with detrimental effects in the host organism, i.e., acute respiratory distress syndrome (ARDS), a form of severe hypoxemic respiratory failure associated with major inflammatory injury to the lung cells and extravasation of protein-rich edema fluid into the airspace [44,45]. Low dose radiation (<0.5 Gy) has been shown to have indeed, in some cases, anti-inflammatory effects and to modulate the immune response, and has even been suggested for treating pneumonia [46]. This LDIR exposure is not expected to exert significant selective pressure on the new coronavirus. Therefore, and based also on recent suggestions, one can hypothesize that a low dose treatment of 30 to 100 cGy to the lungs of a patient with COVID-19 pneumonia could ameliorate the inflammation significantly and relieve the life-threatening systemic symptoms of the infection [47].

## Figures and Tables

**Table 1:** **Coronaviruses under study.** Name, abbreviation, NCBI genome accession code and size (in bp) for each virus are reported.

Family	Name	Abbreviation	NCBI Code
alphacoronavirus	Feline infectious peritonitis virus	FIPV	NC_002306.3
alphacoronavirus	Human coronavirus 229E	HCoV-229E	NC_002645.1
alphacoronavirus	Human coronavirus NL63	HCoV-NL63	NC_005831.2
alphacoronavirus	Miniopterus bat coronavirus 1A	Mi-BatCoV 1A	NC_010437.1
alphacoronavirus	Miniopterus bat coronavirus 1B	Mi-BatCoV 1B	EU420137.1
alphacoronavirus	Miniopterus bat coronavirus HKU8	Mi-BatCoV HKU8	NC_010438.1
alphacoronavirus	Porcine epidemic diarrhea virus	PEDV	NC_003436.1
alphacoronavirus	Porcine respiratory coronavirus	PRCV	DQ811787.1
alphacoronavirus	Rhinolophus bat coronavirus HKU2	Rh-BatCovHKU2	NC_009988.1
alphacoronavirus	Scotophilus bat coronavirus 512	Sc-BatCoV 512	NC_009657.1
alphacoronavirus	Transmissible gastroenteritis virus	TGEV	NC_038861.1
betacoronavirus	Bovine coronavirus	BCoV	NC_003045.1
betacoronavirus	Equine coronavirus	ECoV	LC061274.1
betacoronavirus	Human coronavirus HKU1	HCoV-HKU1	NC_006577.2
betacoronavirus	Human coronavirus OC43	HCoV-OC43	NC_006213.1
betacoronavirus	Mouse hepatitis virus	MHV	NC_001846.1
betacoronavirus	Porcine hemagglutinating encephalomyelitis virus	PHEV	DQ011855.1
betacoronavirus	**Severe acute respiratory syndrome-related coronavirus 2**	**SARS-CoV-2**	NC_045512.2
betacoronavirus	Severe acute respiratory syndrome-related coronavirus	SARSr-CoV	NC_004718.3
betacoronavirus	SARS-related Rhinolophus bat coronavirus HKU3/	SARSr-Rh-BatCoV HKU3	NC_009694.1
betacoronavirus	Middle East respiratory syndrome-related coronavirus	MERS-CoV	NC_019843.3
betacoronavirus	Bat coronavirus HKU9-1	Ro-BatCoV HKU9	NC_009021.1
betacoronavirus	Pipistrellus bat coronavirus HKU5	Pi-BatCoV HKU5	NC_009020.1
betacoronavirus	Tylonycteris bat coronavirus HKU4	Ty-BatCoV HKU4	NC_009019.1
gammacoronavirus	Avian infectious bronchitis virus	IBV	NC_001451.1
gammacoronavirus	Beluga whale coronavirus SW1	SW1	NC_010646.1
gammacoronavirus	Turkey coronavirus	TCoV	NC_010800.1
deltacoronavirus	Bulbul coronavirus HKU11-934	BuCoV HKU11	NC_011547.1
deltacoronavirus	Munia coronavirus HKU13-3514	MunCoV HKU13	NC_011550.1
deltacoronavirus	Thrush coronavirus HKU12-600	ThCoV HKU12	NC_011549.1

**Table 2:** Statistics of SARS-CoV-2.

	A	C	G	U
**ObsN**	12688	7693	8393	13709
**Freq.**	0.30	0.18	0.20	0.32

**Table 3:** Codon usage biases of different coronaviruses under study.

Abbr.	ENC	CAI
BCoV	52.10 ± 2.36	0.69 ± 0.04
BuCoV HKU11	51.41 ± 1.85	0.68 ± 0.04
ECoV	49.31 ± 4.02	0.691 ± 0.02
FIPV	51.56 ± 1.99	0.67 ± 0.048
HCoV-HKU1	44.58 ± 7.33	0.67 ± 0.02
HCoV-229E	50.29 ± 3.62	0.68 ± 0.02
HCoV-NL63	44.67 ± 5.35	0.66 ± 0.03
HCoV-OC43	49.57 ± 3.66	0.692 ± 0.02
IBV	50.65 ± 2.90	0.65 ± 0.05
MERS-CoV	53.08 ± 2.53	0.69 ± 0.03
MHV	53.62 ± 1.72	0.71 ± 0.02
Mi-BatCoV 1A	48.23 ± 3.81	0.68 ± 0.03
Mi-BatCoV 1B	49.31 ± 4.11	0.68 ± 0.03
Mi-BatCoV HKU8	50.12 ± 4.14	0.70 ± 0.02
MunCoV HKU13	53.96 ± 0.86	0.69 ± 0.04
PEDV	52.44 ± 2.153	0.68 ± 0.04
PHEV	51.09 ± 3.553	0.68 ± 0.02
Pi-BatCoV HKU5	53.91 ± 1.36	0.70 ± 0.04
PRCV	51.27 ± 3.15	0.67 ± 0.03
Rh-BatCovHKU2	48.08 ± 4.49	0.70 ± 0.02
Ro-BatCoV HKU9	50.91 ± 2.31	0.68 ± 0.03
SARSr-CoV	53.64 ± 2.43	0.67 ± 0.04
**SARS-CoV-2**	51.98 ± 2.59	0.72 ± 0.05
SARSr-Rh-BatCoV HKU3	54.30 ± 1.61	0.67 ± 0.03
Sc-BatCoV 512	52.38 ± 2.63	0.68 ± 0.04
SW1	50.86 ± 1.791	0.70 ± 0.03
TCoV	51.34 ± 2.31	0.66 ± 0.05
TGEV	51.39 ± 3.27	0.67 ± 0.04
ThCoV HKU12	51.43 ± 2.83	0.68 ± 0.03
Ty-BatCoV HKU4	50.37 ± 3.74	0.68 ± 0.03

**Table 4:** **Parameters of the linear regressions in Forsdyke plots.** None of these plots intersect. The value of each parameter increases from M (lowest) to N (highest). Negative x values indicate flexibility to respond to global pressure to change DNA sequence (virtual time axis) in order to prevent recombination, and to thus allow species divergence (i.e., generate SARS-CoV-2). When recombination is still possible, then two diverging genomes in the same cell will blend, so militating against protein differentiation would occur in time, corresponding to positive x values.

Gene	Y Intercept	Slope	X Intercept
**Matrix (M)**	1.59	0.91	−1.74
**Envelope (E)**	9.87	1.04	−9.46
**Spike surface (S)**	14.95	1.14	−13.15
**RNA replicase (RdRP)**	17.82	1.18	−15.19
**Nucleocapsid (N)**	23	1.30	−17.93
